# High-intensity interval training outperforms moderate exercise to improve aerobic capacity in patients with recent-onset idiopathic inflammatory myopathies: a multicentre randomised controlled trial

**DOI:** 10.1016/j.ebiom.2025.106051

**Published:** 2025-11-27

**Authors:** Kristofer M. Andreasson, Cecilia Leijding, Maryam Dastmalchi, Antonella Notarnicola, Stefano Gastaldello, Takashi Yamada, Heléne Sandlund, Dag Leonard, Håkan Westerblad, Ingrid E. Lundberg, Daniel C. Andersson, Helene Alexanderson

**Affiliations:** a Karolinska University Hospital, Theme Women's Health and Allied Health Professionals, Medical Unit Allied Health Professionals, Stockholm, Sweden; b Division of Rheumatology, Department of Medicine, Karolinska Institutet, Solna, Stockholm, Sweden; c Department of Physiology and Pharmacology, Karolinska Institutet, Stockholm, Sweden; d Department of Gastro, Dermatology and Rheumatology, Karolinska University Hospital, Theme Inflammation and Aging, Stockholm, Sweden; e Graduate School of Biomedical and Health Sciences, Hiroshima University; f Department of Medical Sciences, Section of Rheumatology, Uppsala University, Uppsala, Sweden; g Karolinska University Hospital, Theme Heart, Vascular and Neuro, Cardiology Unit, Stockholm, Sweden

**Keywords:** Aerobic capacity, Exercise, High-intensity interval training, Idiopathic inflammatory myopathies (IIM), Mitochondria, Myositis

## Abstract

**Background:**

To investigate efficacy, safety, and tolerance of high-intensity interval training (HIIT) vs. clinical standard low-moderate intensity home-based exercise (CON) to improve aerobic capacity, muscle endurance, and mitochondrial function in patients with recent onset, idiopathic inflammatory myopathies (IIM).

**Methods:**

Twenty-three patients with recent onset IIM were randomised into HIIT or CON groups. Both groups underwent 12 weeks of exercise training. The HIIT did 3 sessions/week, always supervised during the first three weeks. Then, training was supervised 1–3 session per week based on an individual assessment of the participants’ preference and ability to perform HIIT in the clinic. The CON received one supervised session and then exercised five days per week at home, following clinical standard. Primary outcome was maximal exercise test (VO_2peak_ l/min and ml/kg x min, peak power (Watt), time-to-exhaustion TTE min/sec), with secondary outcomes mitochondrial protein expression in muscle. Safety was assessed by disease activity (serum levels of muscle enzymes, muscle strength (MMT8), Physician Global Assessment, pain, and fatigue (VAS, 0–100).

**Findings:**

HIIT resulted in a 16% increase in VO_2peak_ L/min, significantly higher than the 1.8% change in CON (95% CI 0.1; 0.47). Peak power and TTE improved significantly more in HIIT, 18% and 23%, respectively, compared to CON, 8% and 12% (95% CI 3.9; 30.8 and 00:06; 03:18, respectively). Muscle biopsies (HIIT n = 7, CON n = 6) showed increases (p < 0.05) in central mitochondrial protein expression in HIIT but not CON, suggesting enhanced mitochondrial function. Both groups maintained stable serum muscle enzymes indicating no increase in disease activity from the intervention. Muscle disease activity remained low and unchanged in both groups (95% CI −1.2; 1.0), physician global activity and MMT8 significantly improved within CON (95% CI −1.7; −0.26 and 0.1; 3.9, respectively) but not in the HIIT group.

**Interpretation:**

HIIT is an effective and safe exercise intervention to improve aerobic fitness, muscle endurance, and mitochondrial function in patients with recent onset IIM. This approach should be considered an adjuvant treatment in managing IIM, potentially health-enhancing for these patients.

**Funding:**

10.13039/501100004359Swedish Research Council, the 10.13039/501100007949Swedish Rheumatism Association, Stockholm County Research Grant (ALF), King Gustaf V 80-year Foundation, the Swedish Heart and Lung Foundation, 10.13039/100009389Promobilia Foundation, and Stig Thune Foundation.

**Research in context**

**Evidence before this study**

Exercise is recognised as an essential component in the treatment of idiopathic inflammatory myopathies. While one study has demonstrated that moderate-intensity exercise to be safe in individuals with recent-onset IIM—it did not show superiority over medical treatment alone. Most exercise studies to date have employed light-to moderate-intensity regiments in patients with longstanding disease, in whom chronic pathological changes are already present. Implementing effective exercise interventions early in the disease course may improve outcomes and prevent disability. However, it remains unclear whether high-intensity exercise is tolerated in recent-onset IIM and whether it may provide an effective means of improving exercise tolerance in patient with IIM.

**Added value of this study**

This study demonstrates that high-intensity interval training (HIIT) is a safe and effective exercise intervention for individuals with recent-onset IIM. Compared to standard-of-care exercise training, HIIT is more effective in improving aerobic capacity as indicated by peak oxygen uptake (VO_2peak_), which increased 16% (HIIT) compared to 1.8% after standard-of-care moderate-intensity home exercise. Moreover, HIIT elicited greater exercise-induced adaptations with increased expression of proteins associated to aerobic metabolism mitochondrial function in muscle tissue compared to moderate-intensity exercise.

**Implications of all the available evidence**

High-intensity interval training is a safe, well tolerated exercise intervention for patients with recent onset IIM and should be considered for implementation in clinical practice. The demonstrated safety further the feasibility of introducing individually tailored intensive training programs designed to address patients’ specific needs.

## Introduction

Idiopathic inflammatory myopathies (IIM) are a heterogenous group of autoimmune disorders typically characterised by chronic muscle inflammation. Muscle weakness and reduced muscle endurance as well as systemic features including inflammation in extra-muscular organs such as lungs, skin, heart, and joints are common. In addition, pain and fatigue are frequently reported.^1^ Medical treatment consists of an initially high dose of oral glucocorticoids in combination with steroid sparing agents, such as methotrexate with add-ons depending on treatment response, organ involvement and disease severity. The treatment response and prognosis vary, and although relying on pharmacological treatment, exercise is an adjuvant treatment and imperative to effectively manage IIM.^2^^,^^3^

Patients with IIMs typically suffer from muscle weakness, fatigue, and reduced endurance capacity. At diagnosis, patients have approximately 70% reduced muscle endurance compared to reference values as measured by the Functional Index-2 (FI-2), and 6% reduced maximal strength as measured by the Manual Muscle Test 80 (MMT8), with marginal improvement at 1-year follow-up.^4^ Whether due to reduced maximal muscle strength or reduced muscle endurance capacity, the impaired muscle function is often sustained and rarely recovers to reference values leading to permanent limitations in daily life.

The cause of muscle impairment is less understood but is thought to involve immune and non-immune mechanisms. Although inflammatory cells are often seen in muscle tissue, the level of inflammation in the muscle does not correlate with reduced muscle strength or impaired endurance.^5^^,^^6^ Several mechanisms have been proposed and point to a multifactorial model that includes tissue remodelling (e.g., capillary dysfunction^7^ and fibrosis^8^), immune mediated mechanisms,^9^^,^^10^ and impaired myocyte functions (e.g., contractility, mitochondrial dysfunction and endoplasmic reticulum (ER) stress^11^, ^12^, ^13^). Impaired mitochondrial function might be indicated by the observation of cytochrome C oxidase (COX) negative fibres often observed in muscle biopsies from patients with IIM. Impaired mitochondrial function will be a factor of reduced aerobic capacity as the ability to use oxygen for energy production in the muscle will be limited.^14^ Further, the capillary density can be reduced, specifically in patients with DM, which may reduce oxygen supply to the muscle tissue.^15^^,^^16^ Physical inactivity and side effects of drug treatment may also contribute to muscle weakness.^17^^,^^18^

Maximal oxygen consumption (VO_2peak_) is a critical marker for aerobic fitness that correlates to overall survival and muscle aerobic metabolism.^19^^,^^20^ The VO_2peak_ depends on cardiac output and the peripheral arterio-venous oxygen extraction that depends on the micro circulation and metabolic function in the working muscle (e.g., mitochondrial oxygen consumption). There is a close relationship between VO_2peak_ and muscle mitochondrial function, and VO_2peak_ can effectively be increased with exercise training including high-intensity interval training (HIIT) in healthy individuals.^19^^,^^21^ In patients with IIM (i.e., PM, DM), VO_2peak_ is known to be reduced by approximately 25–50%.^6^^,^^22^ The mechanism of reduced VO_2peak_ in IIM is not clear. Yet, it is independent of the presence of lung diseases in these patients^23^^,^^24^ and likely depends on reduced peripheral O_2_ consumption and/or cardiac output.

Exercise was once considered harmful for patients with IIM due to an imagined risk of aggravated myofibre damage and disease activity. However, this concept has been rebutted by numerous studies that show safety and clinical benefits of mild-to moderate exercise.^25^ Further, moderately intensive endurance exercise can reduce disease activity and inflammation as well as improve mitochondrial enzyme activity in patients with longstanding inflammatory low-active IIM.^22^^,^^26^ Out of the exercise studies published, randomised controlled trial designs are rare.^25^ The only RCT on exercise conducted in patients with recent onset IIM, showed that easy to moderate intensity home exercise in combination with medical treatment is safe but did not outperform medical treatment alone in the non-exercising control group, likely due to the use of low-moderate intensity training protocol.^27^^,^^28^ However, the confirmation of safety of the home exercise program at time of diagnosis has led to the use of this training protocol as standard of care adjunct therapy for patients with IIM in Sweden. However, it is unclear if exercise training on high intensity levels is effective to improve physical capacity without aggravating disease activity, pain, and fatigue levels in patients with recent onset IIM.

Our hypothesis is that exercise on high intensity is an effective intervention to improve aerobic fitness and induce muscle adaptations in IIM. The previously evaluated exercise protocols in IIM have been of too low intensity level to induce adequate physiological adaptations. Moreover, we believe that protocols with continuous and longer exercise sessions, although on lower intensities may be less feasible in recent-onset IIM due to substantial fatiguability seen in this patient group. In contrast, high intensity short interval-based exercise may be more tolerable, render more muscle adaptations and thereby being more effective to improve exercise capacity in patients with recent-onset IIM. We hypothesised that HIIT would be tolerated for patients and lead to increased VO_2peak_. Secondary, we hypothesised that HIIT would induce beneficial adaptations to muscle metabolism in IIM.

This multicentre randomised controlled trial compared 12 weeks of HIIT to the current standard of care, low-moderate intensity home-based exercise. The primary objective was to test the hypothesis that HIIT is more effective to improve aerobic capacity in patients with recent onset IIM, and that it still meets safety standards. In conjunction to this we tested the hypothesis that HIIT may lead to aerobic adaptations in the IIM muscle with increased mitochondrial proteins.

## Methods

This study was approved by the Regional Ethics Review Board in Stockholm (Dnr: 2016/2444-31), who also approved two amendments regarding serum samples and including Akademiska Hospital in Uppsala (Dnr: 2018/1350-32 and 2018/2614-32. The project is registered at ClinicalTrials.gov (ID NCT03324152). All participating patients signed an informed consent before being included. Patient representatives were involved in designing the project plan.

### Patients

All newly diagnosed patients with any type of adult IIM, except inclusion body myositis (IBM), registered at the Karolinska University Hospital in Stockholm between October 2017–April 2023, and at Akademiska Hospital in Uppsala between February 2019 and April 2023, who fulfilled the inclusion criteria, were invited to participate. Specific sex or gender was not considered for in the study design.

The inclusion criteria were probable or definite polymyositis (PM) or dermatomyositis (DM) according to EULAR/ACR 2017 Criteria for IIM,^29^ antisynthetase syndrome (ASyS) according to Connors Criteria,^30^ or immune-mediated necrotising myopathy (IMNM) according to 2016 ENMC Criteria,^31^ 18–70 years old, <12 months of diagnosis duration and medical treatment.

Exclusion criteria were: IBM according to the 2013 ENMC criteria,^32^ active cancer, heart- or lung involvement or disease contraindicating intensive exercise (e.g., severe interstitial lung disease, oxygen supplementation or myocarditis), severe muscle weakness, pain or general fatigue hindering participation in HIIT, and osteoporosis.

**Fig. 1:**
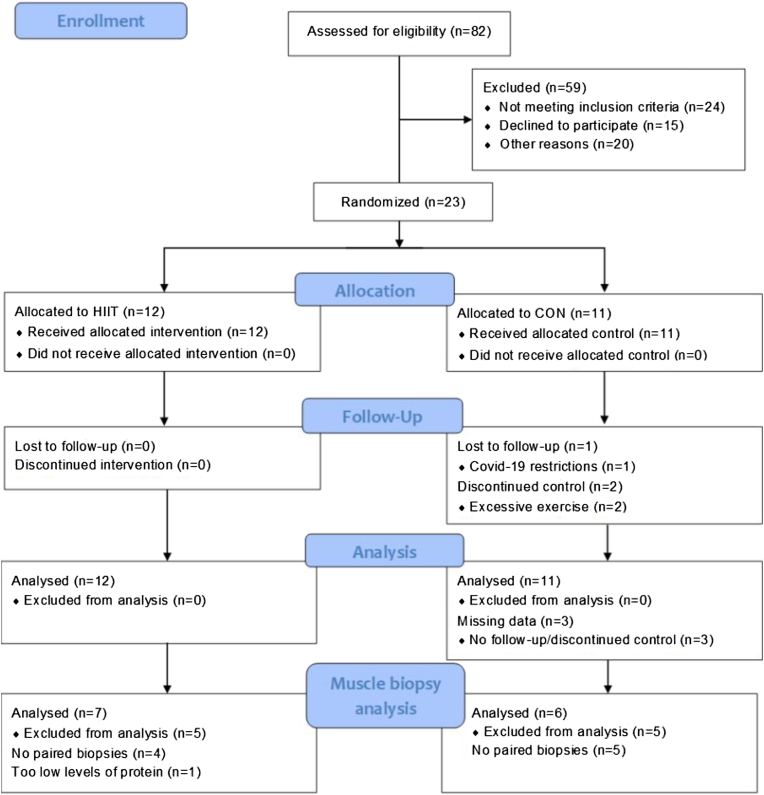
**Recruitment flowchart. HIIT, high-intensity interval training; CON, control group.** Linear Mixed Model including all participants (intention-to-treat) were applied at the stage of “analysis”, while paired T-test with specified included participants were applied at the stage of “Muscle biopsy analysis”.

Twenty-three patients, out of 82 screened, were enrolled - see Table 1 for demographics. Out of the 59 not included, 24 did not meet inclusion criteria, or met exclusion criteria, 15 declined participation and 20 were not included due to other reasons (e.g., not responding calls, not speaking Swedish or English, living in a different region even though diagnosed in our clinic), see recruitment flow chart (Fig. 1).

**Table 1:** Demographics of 23 patients before and after 12 weeks of exercise.

Variables	HIIT		CON	
	Baseline n = 12	12-week follow-up n = 12	Baseline n = 11	12-week follow-up n = 8
Sex^a^ F/M, n	6/6	6/6	9/2	7/1
Diagnosis PM/DM/ASyS, n	4/4/4	4/4/4	0/6/5	0/4/4
Age, years (min–max)	34.5 (24–65)		57 (41–64)	
Weight, kg (IQR)	77 (71.5–83)	78 (72–82)	67 (58–82)	66 (63–76)
Disease duration, months (min–max)	5 (3–11)		5 (1–8)	
Symptom to diagnosis, months (min–max)	10 (1–44)		3.5 (1–17)	
Pulmonary disease activity >0 cm, n^b^	6	3^1^	4^1^	2
Pulmonary disease activity, 0–10 (min–max)	0.45 (0.0–2.5)	0 (0.0–1.8)^1^	0 (0–2.5)^1^	0 (0–3.1)^3^
DMARD AZA/MMF/HYD/MTX/SEND/RIT (n total)	1/5/X/4/X/6 (16)	1/6/X/5/X/5 (17)	X/4/1/3/1/3 (12)	X/4/2/3/X/2 (10)
Prednisone, mg/day (IQR)	10.6 (4.7–22.5)	5 (0–9)	15 (12.5–17.5)^2^	6.2 (3.5–10.5)
CK, μkat/L (min–max)	1.5 (0.6–10.2)	1.5 (0.5–11.3)	1.2 (0.42–2.2)^2^	1.5 (0.42–2.8)^1^
LD, μkat/L (min–max)	3.7 (2.9–6.9)	3.7 (2.4–6.6)	3.7 (3.1–6)^2^	3.7 (2.7–6)^1^
ASAT, μkat/L (min–max)	0.42 (0.26–1.44)	0.41 (0.25–1.97)	0.34 (0.26–1.47)^2^	0.42 (0.32–0.55)^1^
ALAT, μkat/L (min–max)	0.47 (0.13–0.98)	0.40 (0.21–1.23)	0.41 (0.14–0.92)^2^	0.31 (0.25–1.37)^1^
Pain, Visual Analogue Scale, 0-100	14.5 (4–17.5)	10.5 (1.2–19.2)	14.5 (3.5–19)	16.5 (2.2–33.8)
Fatigue, Visual Analogue Scale, 0-100	35 (22.8–61.8)	18 (15–33)	33.5 (15.5–55.8)	25.5 (10.2–43.8)

Data presented in median (interquartile range) except when absolute numbers as specified by n. HIIT, high-intensity interval training; CON, control; F, female; M, male; PM, polymyositis; DM, dermatomyositis; ASyS, antisynthetase syndrome; DMARDs, disease modifying anti-rheumatic drugs; AZA, azathioprine; MMF, mycophenolate mofetil; HYD, hydroxychloroquine; MTX, methotrexate; SEND, sendoxan; RIT, rituximab; mg, milligramme; CK, creatine phosphokinase; μkat/L, microkatals per litre; LD, lactate dehydrogenase; ASAT, aspartate transaminase; ALAT, alanine transaminase. Normative values male/female: CK 0.8–6.7/0.6–3.5, LD < 3.5/<3.5, ASAT <0.76/<0.61, ALAT <1.1/<0.76. 1 μkat/L is equal to 60 IU/L. Superscript numbers indicate n of missing data.

a As registered in the medical record.

b Types of pulmonary diseases: antisynthetase autoantibody associated interstitial lung disease (anti-ARS-ILD), myositis-related nonspecific interstitial pneumonia (NSIP), diffuse alveolar haemorrhage, hilar enlargement, ground glass changes, fibrosis.

If there was an uncertainty of the patient's ability to perform the intensive exercise program, discussion within the medical team consisting of medical doctors (rheumatologists and cardiologist, and other specialities if needed), patient responsible doctor and physical therapists.

### Assessments

#### Primary outcome maximal exercise test

VO_2peak_ L/min was the primary outcome and was assessed by a maximal exercise test performed on a stationary bike using a standardised protocol for all participants. VO_2peak_ was measured breath-by-breath gas exchange with Innocor model INN00500 (COSMED).^33^ First, a resting electrocardiogram (ECG) was registered, then biking started on 30 W with a step increase of 10 W per minute until exhaustion. The participant was instructed to keep cycling until exhaustion, and when unable to keep 60–65 RPM the test was stopped. Staff could stop the test if appropriate due to live-ECG or participant-reported symptoms (e.g., dizziness or chest pain). Every minute blood pressure was measured, and the participants rated the perceived exertion (Bourg's RPE, 6–20), breathlessness and chest pain (Bourg's CR10, 0–10). The test was followed by a recovery of a minimum of 6 min. The primary outcome of this test was VO_2peak_ in litres/minute (L/min), peak load in watts (peak power), peak heart rate (HRpeak), and time-to-exhaustion (TTE), which was the time measured from start to finish of the test (peak power and TTE being measures of muscle endurance). The test was performed approximately one week after the last exercise session and was led by independent staff at the physiology department who were unaware of group allocation; two biomedical scientists, one handling the Innocor assessment and one in charge of the rest of the vtest. A medical doctor was always present or immediately available during the test.

#### Secondary outcomes

##### Biopsy procedure

Muscle biopsies were taken from vastus lateralis or tibialis anterior at diagnosis (PRE) and at follow-up one week post exercise intervention (POST) using the percutaneous conchotome technique under local anaesthesia in the outpatient clinic.^34^ Muscle biopsy tissue was analysed for levels of mitochondrial protein expression (electron transport chain complexes and markers of mitochondrial content). See Supplementary Data S2 for details on methods and procedures.

##### Disease activity and safety

At enrolment the patient visited their treating rheumatologist and the myositis team nurse. Blood samples for serum levels of enzymes (creatine kinase (CK), lactate dehydrogenase (LD), aspartate aminotransferases (AST) and alanine aminotransferases (ALT)) as markers that could indicate muscle damage were taken according to clinical routine. Manual Muscle Test 80 (MMT8) (isometric strength assessment tested where external force is applied by the assessor and strength is rated 0–10 per muscle group tested), the Myositis Disease Activity Assessment Tool (MDAAT) muscle disease activity, and physician global assessment (PhGA)^35^ were assessed using a Visual Analogue Scale 0–100 (VAS). MDAAT muscle disease activity includes muscle inflammation and myalgia, PhGA is a physician judgement of overall disease activity based on all clinical and laboratory assessments. Pain and fatigue (VAS) were assessed, these measures were performed in both groups at baseline and approximately one week 12 weeks after the last exercise session. Before each HIIT session, participants were asked to report any side effects such as delayed onset muscle soreness, pain, fatigue, injuries or falls related to the exercise.

### Exercise protocols

All participants exercised for 12 weeks, and intensity was based on results from the maximal exercise test (HRpeak). To monitor heart rate all participants wore a polar Unite watch and a chest heart-rate monitor (Polar H9) during all exercise sessions. Sessions were then synchronised to PolarFlow.com, to generic user accounts not related to the patient, where compliance as to frequency, intensity (HIIT group reaching >85% and CON not surpassing 70% of HRpeak) and duration of each session was monitored and recorded by the exercise instructor (HA). At least 75% compliance was required for both groups.

#### Intervention group, HIIT

The HIIT protocol consisted of six interval sessions on a stationary bike, three times weekly, with each interval being 30 s long with 2-min-rest of moderate biking in-between. The heart rate was required to reach at least 85% of HRpeak during each interval. Following one test session to identify correct initial exercise load/intensity, the training was ramped up during the first three to four weeks, starting with three sub-maximal intervals, then three maximal, and additional sets were added on subsequently over three to four weeks until reaching the goal of six maximal intervals. Thereafter, the load or time of each interval was increased when needed to ensure that the heart rate exceeded 85%. For participants with severe lower-limb muscle impairments, the protocol was ramped-up by increasing the time of each maximal set with up to 15 s, instead of increasing the load to allow continued biking on a cadence of at least 100 revolutions per minute (RPM). Following the intervals, strength training of shoulders, knee extensors and core was performed on a load of 10 voluntary repetition maximum in one set. All strength training was progressive, e.g., resistance was increased when the exertion was rated as lower than 7 on Bourg CR-10, and the participant could manage to perform correct repetitions. When the participant reached the goal intensity and was familiar with the protocol an individual decision was made for each patient regarding their preferences and ability to perform HIIT at home or in a gym to enhance the feasibility of participation. It was obligatory to perform at least one supervised session in the clinic per week throughout the period. The exercise coach (HA) offered all participants to perform all remaining sessions at the clinic. Compliance and intensity of each set was monitored by a training journal (number of sets, HR/set, leg tiredness and dyspnoea on BORG CR-10, and total exertion on BOR > G RPE 6–20 scale, as well as visual analogue pain and fatigue before and after each exercise session) and wearable HR monitor that allowed supervisors to monitor exercise intensity from a distance. At each clinic-based session, the training journal was evaluated, and participants were asked about their experiences (positive or negative) in relation to their home-based exercise sessions.

#### Comparator group, low-moderate intensive home-based training, CON

This exercise protocol is used as standard for patients with recent onset disease and consisted of eight exercises (step up/walking on the spot for 2 min as a warm-up, shoulder flexion, grip strength squeezing a soft ball, knee extension in a sitting position, hip flexion in a supine position, hip extension by pelvic lifts, sit-ups without neck support and stretching of the exercised muscle groups). Participants should be able to perform ten repetitions per exercise to generate easy to moderate muscle fatigue (3–4 on the BORG CR-10 scale, e.g., moderate—moderate to strong muscle fatigue) and external resistance could be added if necessary.^27^^,^^28^ After one supervised session, participants performed the program five times weekly in combination with a 15-min brisk walk where the participant could not surpass 70% of HRpeak.

### Procedure

The main author (KA), who was not involved in the treatment of patients, scheduled all appointments, supervised the maximal exercise test and was unaware of group allocation. The senior author (HA) was responsible for the exercise, both HIIT and CON, and led the sessions and instructed the participants. Blood tests were taken by the myositis team nurse (HS) who also randomised patients. A randomisation table with blocks of four was used for randomising participants to HIIT and CON, 1:1 and administrated by the objective myositis team nurse. A muscle biopsy was, when possible, taken by one rheumatologist (MD, AN or IL). The same physician also performed the disease activity assessments before and after exercise and were blinded to group allocation. Patient demographics and characteristics were obtained through medical records or the Swedish Rheumatology Quality Registry (SRQ).

### Statistics and data analysis

Data are presented as median (IQR) or exact numbers as appropriate. To analyse within- and between group changes, linear mixed model was used. Changes in mitochondrial enzyme expression were analysed by paired T-tests. Analyses were performed using R and RStudio, see S2 for details (packages used, prototype equation). The training-induced change in VO_2peak_ in the first six participants in each group revealed a difference of 9% (SD = 14%) between the groups. A power analysis based on these values (β = 0.2, α = 0.05) showed that 21 participants were required to detect a statistically significant difference between the two groups. Intention-to-treat analyses were performed for all, but muscle biopsy analysis. To mitigate the issue of missing data, per-protocol analyses were performed as well and are available in Supplementary Data S1 (sheet “Per-protocol data” and “Per-protocol CI”).

### Role of funders

The funders of this study were not involved in the design, data collection, data analysis, data interpretation, or writing of this article in any way.

## Results

### Exercise capacity

**Fig. 2:**
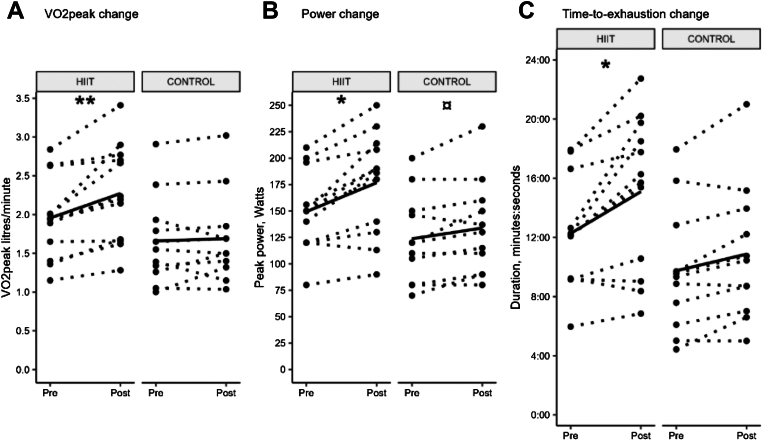
**Results from maximal exercise test.** Change from baseline (pre) and follow-up (post) per group. Dashed lines show change per participant, solid line shows group mean change. All data were analysed with linear mixed model, p-values are used for graphic simplicity, see 95% CI in Table 2. VO_2peak_: peak oxygen consumption; HIIT: high-intensity interval training. ∗ = between-group significance in favour of HIIT (∗ = p < 0.05; ∗∗ = p < 0.01), ¤ = within-group significance. A: Vo2peak change; B: Power change; C: Time-to-exhaustion change.

To compare the effectiveness of the two exercise interventions to improve aerobic fitness, we measured VO_2peak_ at baseline and after 12 weeks. The change in VO_2peak_ (L/min) was significantly larger for the HIIT group with a 16% increase compared to 1.8% for CON (95% CI of 0.10; 0.47; Fig. 2a; Table 2). Furthermore, VO_2peak_ after normalisation to bodyweight (mL/kg/min) was also significantly improved in the HIIT, 16%, compared to the CON group, 4% (95% CI 0.83, 5.10), see Supplementary Table S2; Figure S4. VO_2peak_ relative sex, weight, and age, most participants had a “very low” VO_2peak_ at baseline, indicating a similarly impaired relative aerobic fitness among participants (see Supplementary Data S1, sheet “VO_2_ sex-age-weight”).

**Table 2:** Results of aerobic capacity, muscle strength and disease activity assessments after 12 weeks of exercise.

Variables	HIIT			CON			Between-group change interaction group^a^ time, estimate (95% CI)
	Baseline (n = 12)	12-week follow-up (n = 12)	Within-group change, estimate (95% CI)	Baseline (n = 11)	12-w follow-up (n = 11)^a^	Within-group change, estimate (95% CI)	
VO_2peak_ L/min	1.94 (1.59–2.17)	2.22 (1.68–2.71)	**0.32 (0.18; 0.45)**	1.55 (1.3–1.86)	1.5 (1.36–1.77)	0.03 (−0.1; 0.16)	**0.29 (0.1; 0.47)**
VO_2peak_ mL/kg/min	24.8 (21.4–28.6)	29.2 (25.4–32.5)	**3.92 (2.34; 5.49)**	22.1 (19.3–28.4)	23.4 (19.8–29.8)	0.95 (−0.6; 2.5)	**2.97 (0.83; 5.1)**
Peak power, W	150 (120–166)	188 (138–210)	**27 (18; 37)**	120 (92.5–148)	130 (100–155)	**10 (0.4**; **20.0)**	**17.3 (3.9; 30.8)**
Peak Heart rate	171 (165–187)	172 (162–187)	−2.25 (−6.5; 2.0)	157 (145–172)	161 (149–169)	−0.18 (−4.0; 4.0)	−2.1 (−7.9; 3.7)
Time to exhaustion, mm:ss	12:10 (09:12–13:38)	15:59 (10:11–18:48)	**02:50 (01:38; 04:00)**	09:20 (06:50–11:16)	10:27 (07:51–13:05)	01:07 (−00:02; 02:17)	**01:42 (00:06; 03:18)**
MMT8, 0–80	80 (79–80)^1^	80 (79.5–80)	0.5 (−1.4; 2.4)	76.5 (73.5–80)^1^	80 (78–80)^1^	**0.2 (0.1; 3.9)**	−1.5 (−4.1; 1.1)
Physician Global Activity, VAS 0–10	2.25 (1.5–3.0)	1.2 (1.1–2.1)^1^	−0.64 (−1.30; 0.0)	2.0 (1.6–3.1)^1^	0.9 (0.6–1.5)^3^	−**1.0 (**−**1.7;** −**0.26)**	0.35 (−0.6; 1.2)
Muscle Disease Activity, VAS 0–10	0 (0.00–1.05)	0.4 (0.00–1.25)^1^	0.02 (−0.8; 0.8)	0.7 (0.00–0.95)^1^	0 (0.0–0.3)^3^	0.09 (−0.8; 1.0)	−0.08 (−1.2; 1.0)

All data presented in median (interquartile range). All data analysed with linear mixed model, within-group change for respective group is baseline vs. follow-up, between-group change is HIIT vs. CON. Significant results marked in bold.

HIIT, high-intensity interval training; CON, control; 95% CI, change is presented in 95% confidence interval; VO_2_, peak oxygen uptake; L/min, liters per minute; mL/kg/min, millilitres per kilogramme per minute; W, watts; time to exhaustion, time from start to finish; mm:ss, minutes:seconds; confidence interval for time to exhaustion in minutes:seconds; MMT8, manual muscle test 80; VAS, visual analogue scale.

a Three participants with missing follow-up data were assigned the group mean change as follow-up result.

Peak power was significantly improved in HIIT, 18%, compared to CON, 8% (95% CI 3.9; 30.8); however, the CON showed a significant within-group improvement as well (95% CI 0.4; 20.0), see Fig. 2b; Table 2. The HIIT group improved significantly in TTE during the maximal exercise test compared to the CON, HIIT 23% compared to CON 11.25% (95% CI 6.0; 198.0), see Fig. 2c; Table 2.

Adding age as a covariate, results remained significantly improved for VO_2peak_ and peak power in favour of HIIT. See Supplementary Data S1 (sheet “Age as covariate data” and “Age as covariate CI”).

Heart rate at peak exercise (HRpeak) remained unchanged with respect to intervention in both groups (Table 2).

### Muscle mitochondrial adaptations

**Fig. 3:**
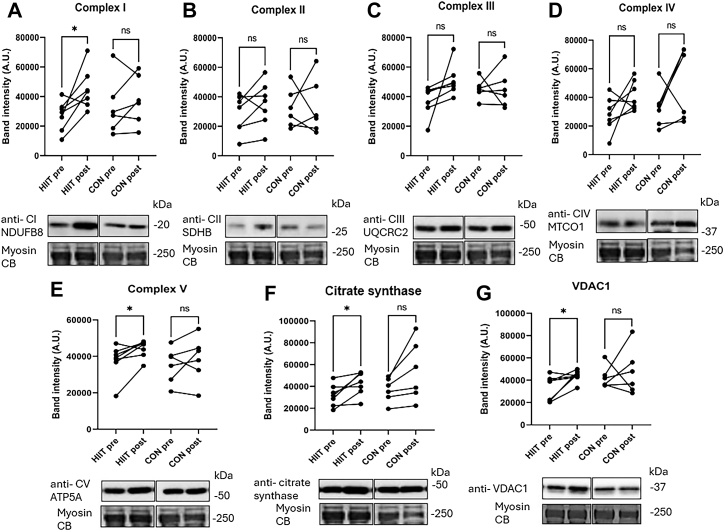
**Increased performance after HIIT is accompanied with increased protein expression in the mitochondrial electron transport chain, citrate synthase (CS) and VDAC1 in skeletal muscle.** Protein quantification for the mitochondria electron transport chain complexes I–V (A–E), citrate synthase (F), and VDAC1 (G) of skeletal muscle lysates from the high-intensity interval training (HIIT) group (n = 7) and the control (CON) group (n = 6), before (PRE) and after (POST) (paired) exercises. Band intensity expressed in arbitrary units (A.U.) were determined by ImageJ and normalised to myosin as internal loading control. Representative blots and the specific antibodies to the quantified proteins: I–V (A–E) anti-OxPhos, anti-citrate synthase (F), and anti-VDAC1 (G) are indicated below the graphs. Paired t-test was performed PRE vs. POST within the groups, ∗p < 0.05. The uncropped western blots are shown in the Supplementary Figure S2.

Aerobic exercise is a potent trigger of muscle adaptations including mitochondrial biogenesis. In conjunction to improved VO_2peak_, we found the muscle expression of mitochondrial respiratory chain complexes I and V (CI & CV), citrate synthase (CS), and voltage-dependent anion channel 1 (VDAC1) increased in the HIIT (p < 0.05), but not in CON. Protein quantification showed higher band intensities for these proteins post-intervention within HIIT but not within CON, indicating enhanced mitochondrial biogenesis and adaptations to aerobic metabolism (Fig. 3).

### Disease activity and safety

At baseline, the HIIT group already had the maximal Manual Muscle Test 80 (MMT8) score of 80 which was significantly better than the CON who displayed close to maximal scores (Table 2). The HIIT group remained at the maximal score after 12 weeks and the CON improved significantly (95% CI 0.11; 3.89). PhGA significantly improved for CON. There was also a tendency towards improvement also in the HIIT group, although not statistically significant. MDAAT muscle disease activity remained unchanged in both groups (Table 2). There was no increase of serum levels of CK, AST or ALT after compared to before the HIIT or CON exercise. At baseline, two participants in HIIT had abnormally elevated CK levels; one remained equally elevated at follow-up, the other had normalised CK at follow-up. LD remained slightly elevated in both groups at all time points (Table 1). There were no statistically significant changes in pain or fatigue (95% CI –37; 10 and −40; 25) as a result of the HIIT. Participants in the HIIT-group reported delayed onset muscle soreness remaining for 1–2 days after each session, also reported instant muscle fatigue subsiding after around 30–60 min after HIIT. No participants reported longstanding pain or fatigue, injuries or falls related to the HIIT. Out of the 23 participants, 20 completed the intervention. Two participants dropped out, and a third could not do follow-up tests due to COVID-19 restrictions, all three randomised to the control group. There was a high compliance with a median 93% of the possible HIIT sessions range 63–100%. For the muscle biopsy analyses, a total of 13 participants were included with sufficient PRE and POST biopsy quality (HIIT n = 7, CON n = 6). Per-protocol analyses, compared to intention-to-treat revealed similar differences in favour of the HIIT except for a significantly higher HRpeak in HIIT compared to CON. For complete per-protocol analyses, age-adjusted results and weight-adjusted VO_2peak_, see Supplementary Data S1.

## Discussion

In this study that investigate efficacy and safety of HIIT compared to a standard-of-care exercise in recent onset IIM using a randomised controlled design, we found that HIIT was more effective compared to current standard-of-care low-moderate exercise interventions to improve aerobic exercise capacity and muscle mitochondrial adaptations. Moreover, the results provide evidence that HIIT is safe and well tolerated in recent onset, IIM.

A notable finding was the significant increase in VO_2peak_ in the HIIT group compared to the CON group. An increase >10% in VO_2peak_ is considered clinically relevant.^36^ In our study, the HIIT group improved VO_2peak_ by 16% corresponding to a clinically significant increase in VO_2peak_ of 4.4 ml/kg/min. In contrast, the CON did not achieve statistically significant or clinically relevant change. Our results from the HIIT intervention are in line with effects of HIIT in the general population, where an average increase of 3.9 ml/kg/min was recently reported in a meta-analysis.^37^ The 4.4 ml/kg/min increase in the HIIT group is a substantial improvement exceeding 1 metabolic equivalent (MET) and underscores the efficacy of HIIT in enhancing aerobic capacity. This magnitude of increased VO_2peak_ is known from other studies to associate with reduced all-cause mortality in healthy men and women.^38^

Almost all participants in our study had a baseline VO_2peak_ corresponding to “very low” aerobic capacity underlining the poor aerobic capacity in patients with IIM.^36^ Yet our HIIT protocol successfully improved aerobic capacity for all participants regardless of initial fitness level. To optimise adherence to the HIIT protocol also for unfit individuals, the initial exercise intensity was set low, based on the HRmax achieved at the baseline VO_2_ test, with gradual progression towards the goal intensity. Aerobic capacity is strongly correlated to self-reported physical health in IIM,^39^ which highlights the importance of assessing and targeting aerobic capacity in an exercise intervention for people with IIM. In the setting of cardiovascular diseases, improved aerobic capacity is strongly associated with reduced progression of cardiovascular disease and improved survival.^40^ To our knowledge it has not yet been studied how aerobic capacity links to disease outcome and mortality in IIM.

In line with VO_2peak_, TTE and peak power showed significant improvements in the HIIT group compared to the CON. HIIT-participants biked 23% longer and achieved an 18% increase in peak power, compared to 12% and 8% in the CON group, respectively. HRpeak did not change in either of the groups, which was expected since maximum heart rate often do not change with increased exercise capacity. Yet, it suggested that the participants reached near peak capacity at both baseline and follow-up.

Mitochondria is the central organelle for aerobic metabolism, electron transport chain, and many other metabolic processes in the muscle. Therefore, it is not surprising that mitochondrial dysfunction is linked to many diseases including IIM.^41^ It is well known that exercise training is a powerful signal for mitochondrial biogenesis in skeletal muscle^42^ and, moreover, that VO_2peak_ is associated to mitochondrial content in the muscle.^43^ In line with this, we found that repeated skeletal muscle biopsies revealed significant increases in mitochondrial protein expression following the HIIT intervention but not in CON. Increased level of expression was revealed in electron transport chain (complex I and IV) proteins and for general markers of mitochondrial content (VDAC and citrate synthase). These proteins are crucial for mitochondrial function. The larger protein expression post-intervention in HIIT than CON, suggests that HIIT more effectively promotes mitochondrial adaptations in muscle, which may contribute to the observed improvements in aerobic capacity and muscle endurance. However, to what extent the increased VO_2peak_ is related to the seen increase in mitochondria is uncertain, as VO_2peak_ is a compounded readout that also depends on haemodynamic and microvascular adaptations. Yet, the molecular adaptations that we see in the muscles are consistent with previous research showing that intensive exercise promotes both muscle mitochondrial function, VO_2peak_^20^ and overall metabolic health in healthy individuals and in patients with IIM.^22^^,^^41^^,^^44^^,^^45^ Our data indicates that the IIM muscles after HIIT, in principle, should have increased capacity to consume oxygen and produce ATP via mitochondrial respiration during exercise.

We also assessed disease activity by measures of serum levels of enzymes found in muscle as surrogate markers of tissue damage (CK, LD, AST, ALT), as well as the PhGA of disease activity and muscle disease activity. While CK levels were not elevated on a group level, LD was consistently elevated for both groups, suggesting ongoing muscle damage or stress. However, LD remained unchanged, suggesting no increased inflammation from either HIIT or CON supporting the safety of HIIT. As expected, HIIT resulted in acute, short-term physical fatigue and one to two days of delayed-onset muscle soreness, however not limiting continuation of HIIT three days per week. Interestingly, the CON group showed a significant improvement in PhGA. Further, CON also had a slight room for improvement in MMT scores which HIIT did not as they had maximal scores already at baseline. Muscle disease activity was scored slightly higher, although non-significant, for the HIIT group at follow-up. However, the change from 0 cm to 0.7 cm is within the error of measurement and probably not clinically relevant. Therefore, our results are consistent with previous studies on exercise in IIM^25^; as there were no signs of increased inflammation or disease activity, suggesting HIIT being a safe intervention also in early phases of disease.

The HIIT group had a maximum score of 80 on MMT8 and thus there was no room for improvement. The CON had almost 96% of maximal score at baseline and improved significantly, after 12 weeks. Overall, the clinical relevance or importance of MMT8, with its previously described ceiling effects,^4^^,^^46^ can be discussed as it might lead to overseen limitations in muscle function in patients. Unfortunately, we were not able to perform FI-2, as it would have added another separate visit to the clinic. However, the results of MMT8 indicate no worsening of muscle strength further underscoring the safety of HIIT.

Despite the rigorous and intensive nature of HIIT, compliance was high, and the protocol was well-tolerated by participants. This is encouraging, as it demonstrates the feasibility of the HIIT in a clinical setting for patients with IIM. The use of heart rate monitors and careful supervision ensured that participants adhered to the prescribed intensity levels, minimising the risk of adverse events. To enhance the feasibility of the HIIT-protocol, participants were free to perform 1–2 sessions in a gym of their choice e.g., if they lived far away from the clinic. Three participants in the HIIT group travelled abroad for one week (for work or vacation) during the 12-week exercise period. However, they maintained their exercise regimens and were monitored on distance with real-time feedback from the exercise instructor. In these cases, rating of pain and fatigue was not always followed as instructed.

The study had some limitations, including missing data for some participants. However, the use of intention-to-treat and per-protocol analyses helped mitigate these limitations. Furthermore, the blinded randomisation and use of a control group strengthens our results. Despite randomisation in blocks of four there was a trend of skewness, yet non-significant, in age, sex, and type of IIM between the two groups (Table 1), which could be a result of the small sample size. The sample size was estimated for the primary outcome VO_2peak_, and the lack of statistically significant differences in some secondary outcomes may be due to lack of statistical power. As IIM is a very rare disease, including large numbers of patients constitutes a logistic challenge. However, we would argue that larger studies that includes patients from multiple clinical centres may be warranted to further validate the efficacy of HIIT in IIM.

The study was initiated as a pilot study since this type of intensive exercise had never been evaluated in patients with recent onset IIM. COVID-19 restrictions forced us to change the inclusion criteria of time from diagnosis from 6 months to 12, to be able to include four patients that were about to start exercise at the time of initiating pandemic restrictions. Further, the pandemic hindered us from including at least two patients with higher disease activity and organ involvement as they were “high-risk patients” and were not allowed into the hospital other than for clinically necessary appointments. Since VO_2peak_ assessments have not previously been conducted in patients with recent onset IIM, participants’ symptoms of exhaustion and failure to continue biking at the pre-specified cadence defined as the upper limit of exercise capacity. In this study we were not able to retrieve paired biopsies from all participants. Initially, this was due to staff shortage and a large re-organisation to a newly built hospital with a lack of routines. Later, it was also due to a freezer breakdown leading to thawed biopsies, or due to biopsy content dominated by fat and too little muscle tissue for the analyses.

### Conclusions

This study indicates that HIIT is more effective compared to standard low-moderate intensity home-based exercise for improving aerobic capacity, muscle endurance, and mitochondrial adaptation in patients with IIM with short disease duration. These findings support the use of HIIT as a safe and effective adjuvant treatment in the management of IIM, which provides an effective strategy to enhance physical fitness and potentially improve quality of life for these patients. Future studies should aim to explore long-term effects of HIIT on both physical fitness, disease activity and possibly the role of HIIT to prevent comorbidities and mortality. Additionally, ongoing investigation of underlying mechanisms of HIIT's impact on inflammation and mitochondrial function could provide valuable insights into the pathophysiology of IIM.

## Contributors

Conceptualisation: H Alexanderson, DC Andersson, IE Lundberg, H Westerblad.

Data curation: KM Andreasson, H Alexanderson, C Leijding, DC Andersson.

Formal analysis: KM Andreasson, H Alexanderson, C Leijding, DC Andersson.

Funding acquisition: H Alexanderson, DC Andersson, IE Lundberg.

Investigation: KM Andreasson, H Alexanderson, C Leijding, DC Andersson, IE Lundberg, A Notarnicola, M Dastmalchi, S Gastaldello, H Sandlund, D Leonard.

Methodology: H Alexanderson, DC Andersson, IE Lundberg, H Westerblad, S Gastaldello, T Yamada.

Project administration: KM Andreasson, H Alexanderson.

Supervision: H Alexanderson, DC Andersson.

Visualisation: KM Andreasson, C Leijding.

Writing–original draft: KM Andreasson, H Alexanderson, DC Andersson, C Leijding.

Writing–review & editing: KM Andreasson, H Alexanderson, DC Andersson, C Leijding, IE Lundberg, H Westerblad, S Gastaldello, T Yamada, D Leonard, M Dastmalchi, A Notarnicola, H Sandlund.

All authors have read and approved the final version of the manuscript.

Data have been accessed and verified by KM Andreasson, C Leijding, DC Andersson, and H Alexanderson.

## Data sharing statement

Data will be provided upon reasonable request as the data set is still being analysed for further publications. To request data, please contact KM Andreasson at Kristofer.andreasson@ki.se.

Code, software, and packages used for analyses are presented in Supplementary Data S2. Further code will be shared upon reasonable request as analyses for other publications have been made in the same workspace.

## Declaration of generative AI and AI-assisted technologies in the writing process

During the preparation of this work the author(s) used ChatGPT from OpenAI in order to improve the language (grammar and readability). After using this tool/service, the author(s) reviewed and edited the content as needed and take(s) full responsibility for the content of the published article.

## Declaration of interests

Kristofer M. Andreasson—have received research funding from Stig Thune foundation and travel grant from the Swedish Heart and Lung Foundation.

Cecilia Leijding—have received travel grant from Scandinavian Physiology Society and Karolinska Institutet.

Maryam Dastmalchi—nothing to declare.

Antonella Notarnicola—nothing to declare.

Stefano Gastaldello—nothing to declare.

Takashi Yamada—nothing to declare.

Heléne Sandlund—nothing to declare.

Dag Leonard—have received funding from Astra Zeneca for research collaboration and personal reimbursement.

Håkan Westerblad–Member of their Scientific Advisory Board at Myocene SA, Liege, Belgium and receives monthly personal allowances. They sell equipment for force measurements. This equipment was not used or in any way related to the present study. Inventor of Impact alert system and method, US Patent 8,742,910. Patent not in any way related to the present study. Editor in Chief, European Journal of Applied Physiology, 2012–2025.

Ingrid E. Lundberg—have received funding from the Swedish Research Council, institutional grants from the Swedish Heart and Lung Foundation, Stockholm County research grant (ALF), Swedish Rheumatism Association, King Gustaf V 80-year foundation, Janssen Pharmaceutica NV, and Astra Zeneca. Consulting fees paid to the institution from Chugai and Novartis. Consulting fees paid to person from Pfizer. Speaker honoraria from Janssen Pharmaceutica NV. Advisory- or Data Safety Monitoring board, personal reimbursement from Merck Healthcare KGaA data Safety monitoring and Pfizer, institutional reimbursement from Chugai, EMD Serono Research & Development Institute, Argenx, Galapagos, and Bristol Myers Squibb. Stocks in Novartis and Roche.

Daniel C. Andersson—have received research grants from King Gustaf V 80-year foundation, Promobilia Foundation, Stockholm County research grant (ALF), and the Swedish Heart and Lung Foundation. Speaker's honoraria from Bristol Mayer Squibb, unrelated to this study. Patent submitted (UK) for the repurposing of a pharmaceutical compound for use in muscle weakness in inflammation and myopathy. Advisory board member in Anacardio, terminated 2022.

Helene Alexanderson—have received research grants from the Swedish Research Council, the Swedish Rheumatism Association, Promobilia Foundation. Unpaied participation in The Myositis Association Advisory Board, and The Swedish Myositis Association Board. Unpaid leadership role in IMACS Rehabilitation and Exercise Scientific IG, MIHRA board member, Swedish Myositis Register board.

## Figures and Tables

**Supplementary Figure S2 and Western Blot:**
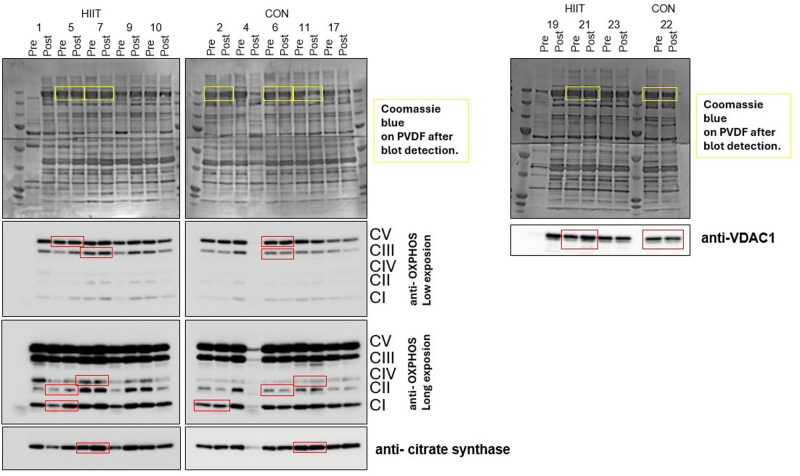
Uncropped immunoblots showing the antibodies used to detect the indicated proteins. The membranes were stained with Coomassie Blue to confirm equal protein loading. A total of 15 μg of skeletal muscle lysate from each sample was loaded per lane and separated by SDS–PAGE. Red boxes indicate the proteins of interest, and yellow boxes denote the myosin band used for loading normalisation, as shown cropped in Fig. 3. Samples ID1 and ID4 were analysed on a separate blot due to partial protein degradation, and ID19 was excluded owing to degradation of the PRE sample.

## Appendix A Supplementary data

Supplementary Figure S1 Change of Vo_2peak_ in mL/kg/min. From baseline (pre) to follow-up (post), adjusted for weight. Dashed lines show change per participant, solid line shows group mean change. All data were analysed with linear mixed model, p-values are used for graphic simplicity, see 95 % CI in Table 2. VO_2peak_: peak oxygen consumption; HIIT: high-intensity interval training; mL: millilitres; kg: kilogramme; min: minute.
