# Supplementary figures and images for: High-intensity interval training outperforms moderate exercise to improve aerobic capacity in patients with recent-onset idiopathic inflammatory myopathies: a multicentre randomised controlled trial
Source: eBioMedicine. 2025 Nov 27;122:106051. doi: 10.1016/j.ebiom.2025.106051 (PMC12703967; doi:10.1016/j.ebiom.2025.106051)

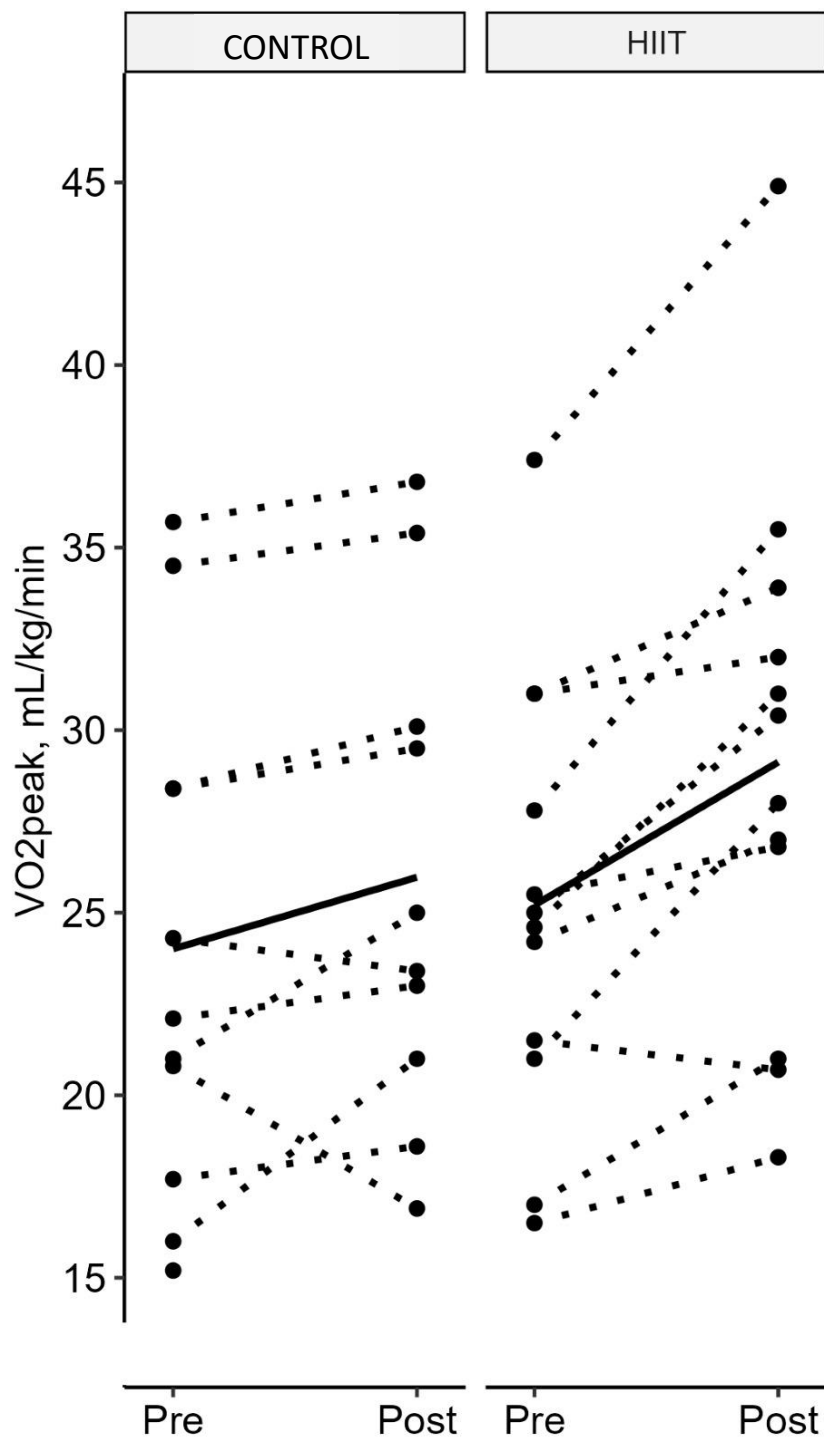

Supplement: Supplementary Figure S1 — Change of Vo2peak in mL/kg/min. From baseline (pre) to follow-up (post), adjusted for weight. Dashed lines show change per participant, solid line shows group mean change. All data were analysed with linear mixed model, p-values are used for graphic simplicity, see 95 % CI in Table 2. VO2peak: peak oxygen consumption; HIIT: high-intensity interval training; mL: millilitres; kg: kilogramme; min: minute. [file mmc1.pdf]
